# Autophagy in Extracellular Matrix and Wound Healing Modulation in the Cornea

**DOI:** 10.3390/biomedicines10020339

**Published:** 2022-02-01

**Authors:** Duraisamy Kempuraj, Rajiv R. Mohan

**Affiliations:** 1 Harry S. Truman Memorial Veterans’ Hospital, Columbia, MO 65212, USA; duraisamyk@missouri.edu; 2 One-Health Vision Research Program, Departments of Veterinary Medicine & Surgery and Biomedical Sciences, College of Veterinary Medicine, University of Missouri, Columbia, MO 65212, USA; 3 Mason Eye Institute, School of Medicine, University of Missouri, Columbia, MO 65212, USA

**Keywords:** autophagy, corneal wound healing, extracellular matrix, fibrosis, inflammation, mast cells, myofibroblast, transforming growth factor-beta 1

## Abstract

Autophagy is a robust cellular mechanism for disposing of harmful molecules or recycling them to cells, which also regulates physiopathological processes in cornea. Dysregulated autophagy causes inefficient clearance of unwanted proteins and cellular debris, mitochondrial disorganization, defective inflammation, organ dysfunctions, cell death, and diseases. The cornea accounts for two-thirds of the refraction of light that occurs in the eyes, but is prone to trauma/injury and infection. The extracellular matrix (ECM) is a noncellular dynamic macromolecular network in corneal tissues comprised of collagens, proteoglycans, elastin, fibronectin, laminins, hyaluronan, and glycoproteins. The ECM undergoes remodeling by matrix-degrading enzymes and maintains corneal transparency. Autophagy plays an important role in the ECM and wound healing maintenance. Delayed/dysregulated autophagy impacts the ECM and wound healing, and can lead to corneal dysfunction. Stromal wound healing involves responses from the corneal epithelium, basement membrane, keratocytes, the ECM, and many cytokines and chemokines, including transforming growth factor beta-1 and platelet-derived growth factor. Mild corneal injuries self-repair, but greater injuries lead to corneal haze/scars/fibrosis and vision loss due to disruptions in the ECM, autophagy, and normal wound healing processes. Presently, the precise role of autophagy and ECM remodeling in corneal wound healing is elusive. This review discusses recent trends in autophagy and ECM modulation in the context of corneal wound healing and homeostasis.

## 1. Introduction

Autophagy is a self-degradative, robust mechanism for disposing of and recycling defective intracellular structures and substances from the cells. It is implicated in cytoprotection, tissue development, tissue plasticity, response to infections, diseases, injuries, regulation of the immune system, and maintaining cellular and tissue-specific microenvironments and functions [1,2,3,4,5]. Autophagy involves extensive or selective degradation, transportation, and degradation of dysfunctional membranes, proteins, invaded pathogens and organelles, as well as the recycling of cytoplasmic components [6]. Macroautophagy, microautophagy, and chaperone-associated autophagy are three distinct types of autophagy; the former is generally termed as autophagy (autophagocytosis) [6,7]. In selective autophagy, specific receptors are involved in the selection of materials for the degradation process. Autophagy constantly captures aged or damaged cellular materials for lysosomal degradation and recycling to maintain cellular and tissue functions [8]. Defective, insufficient, and delayed autophagy leads to reduced degradation, abnormal intracellular protein accumulation, mitochondrial dysfunctions, physiological disturbances, immune dysfunction, ocular/corneal disorders, neurodegenerative disorders, cardiovascular diseases, cancer, renal diseases, musculoskeletal diseases, hepatic disorders, pulmonary disorders, infectious diseases, reproductive dysfunctions, and accelerated aging [1,9,10,11]. 

Autophagy is implicated in most cellular stress-response pathways, including immune responses and inflammatory pathways, as well as ocular diseases [7,12,13,14]. The cornea is an avascular and transparent layer that covers the front of the eye and is directly exposed to the external atmosphere; therefore, it is highly prone to infection and injury. The corneal wound healing process is highly complex, involving multiple cell types, cytokines and growth factors, as well as autophagic mechanisms [9,13,15,16]. Autophagy regulates the homeostasis of the tissues and their functions by regulating matrix deposition and wound healing pathways. 

The extracellular matrix (ECM) is a multifunctional, noncellular, dynamic, and macromolecular complex present in all tissues which is constantly remodeled through ECM producing cells (epithelial cells, fibroblasts, endothelial cells, immune cells), degrading enzymes, and their specific inhibitors [17]. Over 300 ECM components with specific functions have been reported [18]. The ECM is tissue-specific, heterogeneous, and varies from physiological state to disease state [19]. It is a supportive structure/scaffold for cells and tissues, offering tensile strength and preventing overstretching of tissues. The ECM interacts with various cells and regulates cellular and organ functions, survival, migration, differentiation, organ structure, tissue regrowth/regeneration, tissue repair, and scar formation, as well as controlling many biological activities and maintaining a normal tissue-specific microenvironment [17,20]. However, dysregulation of ECM components, structure, stiffness, and abundance is associated with disease pathogenesis, chronic wound/ulcers, and fibrosis, and scar formation. Chronic or severe tissue damage can cause extensive ECM production, and abnormal deposition with insufficient degradation leads to fibrosis [20]. A better understanding of autophagy and ECM regulation in pathophysiological conditions will help in the development of new therapeutics in regenerative medicine and corneal disorders [20,21,22,23,24]. Corneal transparency is maintained by the precise organization and orientation of the collagen fibrils of the ECM. Altered ECM deposition/degradation changes normal corneal function and induces corneal disease pathologies, including scarring that affects corneal transparency [25,26,27]. 

Wound healing is a complex, dynamic, and highly coordinated process of tissue protection involving immune cells, cytokines, chemokines, proteases, and growth factors that repair damaged tissue efficiently [23,24,25]. However, abnormal, prolonged or poor wound healing leads to infections, ulcers, hypertrophic scarring, and chronic wounds [28,29]. The cornea is a highly specialized, transparent, powerful refractive surface and a robust outer barrier protecting ocular tissue. Active military service members, veterans, and civilians show various corneal injuries due to exposure to toxic gases such as sulfur mustard gas, hydrogen sulfide, and chlorine, as well as combat blasts, blast waves, infections, trauma, and traumatic brain injury (TBI)/polytrauma [30,31,32,33,34,35]. Novel therapeutic targets and approaches are needed to improve the corneal wound healing process to maintain sharp vision [16,21,22,36,37]. The ECM can regulate autophagy in normal wound healing mechanisms [28]. The corneal wound healing mechanism is a highly coordinated procedure associated with the cell death/necrosis/apoptosis, migration, proliferation, and differentiation of cells during the ECM remodeling process [38]. The effect of delayed or dysregulated autophagy and ECM modulation in corneal wound healing is not currently well understood. Novel therapeutic approaches could be developed by targeting mechanistic regulation of autophagy affecting corneal wound healing and homeostasis [9,39,40]. This review highlights recent trends in autophagy and ECM modulation in corneal wound healing and homeostasis. 

## 2. Autophagy

**Figure 1:**
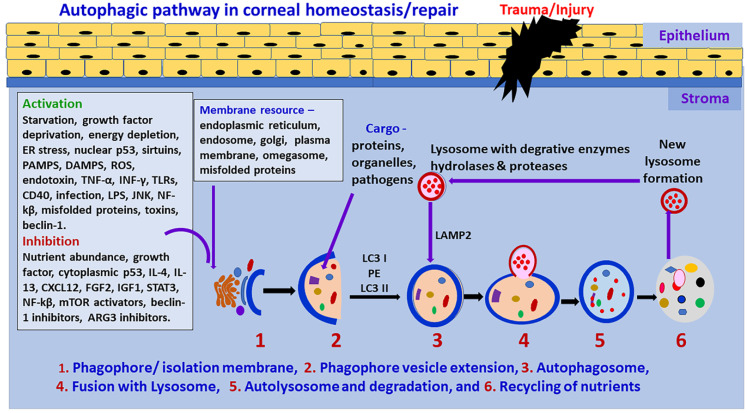
Schematic diagram of the autophagic pathway. Cellular stress, deficiency of nutrients, cytokines and chemokines can initiate autophagic mechanisms. Autophagy begins the initiation stage with the formation of the phagophore. Autophagy is negatively regulated by mTOR and positively regulated by AMPK. Activation of autophagy requires the formation of the ULK1 and phosphorylation of PI3KC3. This activates local phosphatidylinositol-3-phosphate (PI3P) production and nucleation of omegasome. PI3P effectors, including WIP12, are then recruited and interact with ATG-7-ATG12-ATG5-ATG16L1-ATG3 LC3B-conjugation system. This complex, through ATG3, mediates phosphatidylethanolamine (PE) lipidation of ATG8 family proteins such as LC3B, enabling their recruitment to the phagophore membrane. Membranes from various cells, endosomes, mitochondria, ATG9A-containing vesicles exported through AP-4 can contribute to phagophore elongation. The expansion of the double membrane of the phagophore forms a fully closed mature autophagosome. Autophagosomes can engulf intracellular cytoplasmic contents. The fusion of the mature, double membrane autophagosome with a lysosome is enhanced by the SNARE and HOPS system. Fully formed autophagosomes fuse with lysosomes and form autolysosomes. The sequestered organelles, substances, and nutrients are degraded in the autolysosome in the degradation stage by lipases and proteases, and then amino acids and lipids are available for reuse in the cell. The autolysosome is then recycled to form a new lysosome. LPS = Lipopolysaccharides; TLRs = Toll-like receptors.

Autophagy is a degradation and recycling process in cells which is necessary for cell death as well as cell survival in tissues including ocular/corneal cells in ocular homeostasis [3,15]. It plays an essential role in cell survival and maintenance by recycling cellular breakdown products [3,41]. Mitochondria, Golgi, and nuclei form double-membrane vesicles, termed as autophagosomes, which fuse with lysosomes for the degradation of substances [42]. Usually, the mTOR (mammalian Target of Rapamycin) complex suppresses autophagy by ULK1 (inhibiting serine/threonine kinase unc-51-like kinase 1) activity. Intra- and extra- cellular stress, oxidative stress, endoplasmic reticulum stress, starvation, growth factor deprivation, hypoxia, infections (viruses, intracellular bacteria), toxins, certain cytokines/chemokines (tumor necrosis factor-alpha (TNF-α), interferon-gamma (IFN-γ)), sirtuins, immune signals (pathogen-associated molecular patterns (PAMPS), reactive oxygen species (ROS) damage-associated molecular patterns (DAMPS)), inflammation, misfolded proteins, c-Jun N-terminal kinases (JNK), nuclear factor kappa B (NF-kβ), p53 (nuclear), cell surface receptors such as the cluster of differentiation 46 (CD46) and CD40 can initiate autophagy (initiation stage) by stimulating UIk1 kinase and the phosphorylation of its substrate, autophagy-related proteins (ATG 13) and FIP100, as shown in Figure 1 [12]. 

Autophagy initiation is suppressed by nutrient abundance, cytokines such as interleukin-4 (IL-4), IL-13, STAT3 (signal transducer and activator of transcription 3), mTOR activators, Beclin 1 inhibitors, and ATG3 inhibitors. Rapamycin removes the mTOR-associated suppression of autophagy. In the nucleation stage, the formation of PtdIns3P through Vsp34 causes the attraction of more factors. The extension and closure of autophagosome depend on the Atg5/Atg12 conjugate complex, which accelerates the lipidation of LC3 (microtubule-associated protein 1A/1B-light chain 3) to LC3II by phosphatidylethanolamine (PE) [10]. In contrast, autophagy is regulated by mTOR and directly controlled by AMPK (adenosine monophosphate-activated protein kinase). Activation of autophagy is associated with the generation of the ULK1 and PI3K protein systems. At the beginning of autophagy, cytoplasmic targets, including damaged organelles, are separated and contained in a double-membrane structure called a phagophore. Membranes from cells, endosomes, mitochondria, and ATG9A-associated vesicles are transferred via AP-4, and can contribute to the elongation of the phagophore. The expansion and maturation of the phagophore produce an autophagosome. The maturation of the autophagosome involves the transformation of cytosolic protein LC3-I to lipidated membrane-connected LC3-II after its entry into the phagophore membrane. Autophagosomes can engulf defective intracellular cytoplasmic contents. 

The fusion of the fully developed autophagosome with lysosome occurs through SNARE (synaptic-soluble *N*-ethylmaleimide-sensitive factor attachment receptor) and the HOPS complex. Mitochondria, Golgi and nuclei form autophagosome double-membrane vesicles which fuse with lysosome in the degradation process [42]. The targeted/sequestered materials are degraded in the autolysosome in the autophagy degradation stage [6,43]. The autolysosome is then recycled to form a new lysosome; this cycle continues for tissue homeostasis [42]. Metabolic byproducts released from the digestion of cellular materials are used to fuel mitochondria for energy storage in the cells [44]. 

Autophagy is implicated in neutrophils, eosinophils, mast cells, and natural killer cells [2]. Autophagy is constitutively induced in mast cells; however, the exact function of autophagy in mast cells is not fully understood. Autophagy enhances the ability of neutrophils to fight against pathogens and increases their phagocytosis activity, degranulation and IL-1β secretion, as well as the degranulation and release of mediators in mast cells during immune response [2]. LC3-II (an autophagosome marker) and CD63 (lysosomal secretion marker) are located in the cytoplasmic granules of mast cells [45,46]. ATG proteins are crucial in neutrophils and natural killer (NK) cell differentiation [2]. ATG5 primarily involves neutrophil differentiation and mTOR control neutrophil extracellular trap (NET) formation [2,47]. Autophagy is implicated in monocyte differentiation to macrophage and LC3/LC-II-mediated phagocytosis [2]. Blocking autophagy/LC3 or Beclin 1 increases activation of caspase 1, IL-1β and IL-18 release from monocytes/macrophages [2]. Autophagy regulates T cell functions such as survival, signaling and effector functions, as well as the formation of memory T cells [48].

Autophagy is necessary for maintaining immune privilege in the eye [49]. ATG proteins are highly expressed constitutively in the cornea, lens, retina, and orbit in the eye [13]. Autophagy is implicated in the regulation of innate as well as adaptive immune systems by controlling the release of several mediators and inflammasome pathways. Autophagy modulates the inflammatory responses, synthesis and secretion of various inflammatory mediators, and these mediators, in turn, regulate autophagy pathways [50,51,52]. An innate immune response can activate the autophagic pathways to protect the host [53]. Both innate and adaptive immunity is dampened in ocular tissues to maintain immune privilege and prevent excessive inflammatory reactions that could impair vision. However, the loss of immune privilege results in many ocular pathologies [43]. The removal of autophagy genes in the macrophages causes ocular pathologies such as uveitis and blindness by activating inflammasome-associated IL-1β secretin and increasing the severity of the disease [43]. It is known that T helper type 1 (Th1) cytokines transforming growth factor-beta (TGF-β), IL-1, TNF-α, interferon-gamma (IFN-γ), IL-6, and IL-2 can stimulate autophagy pathways; however, Th2 cytokines IL-4, IL-10, and IL-13 can suppress autophagy [50]. Components of autophagic pathways are constitutively expressed in high levels in the cornea, lens, retina, and orbit in the eye. The activation of autophagy is involved in the pathogenesis of ocular disorders, including corneal disorders [13]. Corneal cells such as epithelial and endothelial cells, as well as stromal fibroblasts, express autophagic markers such as LC3/Atg8, Atg5, LAMP1 (lysosomal-associated membrane protein 1), DRAM1 (DNA damage regulated autophagy modulator 1), SQSTM1 (sequestosome 1)/p62, BECN1 (beclin 1)/Atg6, and PRKN2 (E3 ubiquitin-protein ligase parkin) [9,15,42]. Increased expression of autophagic proteins such as LC3A/B and LAMP-1 in corneal fibroblasts has been reported during corneal wound healing. Abnormal corneal wound healing shows the persistence of many myofibroblasts with excess and abnormal ECM deposition in the stroma. Autophagy may help in decreasing or removing excess ECM to prevent fibrosis in the stroma. 

## 3. Extracellular Matrix 

**Figure 2:**
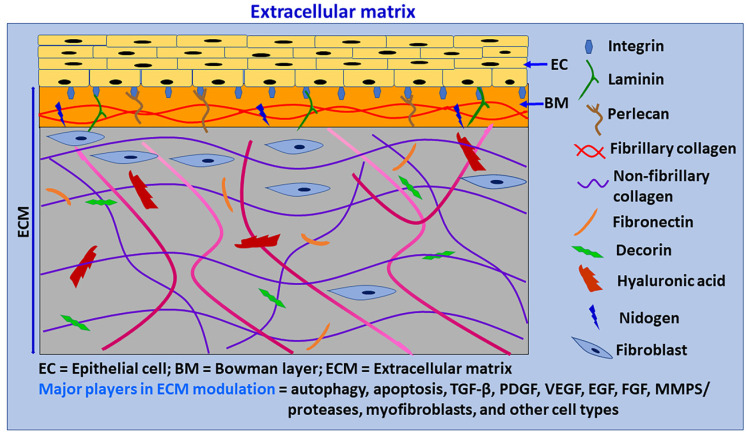
Schematic diagram of primary components of ECM. ECM is a complicated network of proteins that provides a support structure for cells and also interacts with them to modulate their phenotypes, survival, proliferation, migration, differentiation, functions, and apoptosis. ECM is continuously renewed and reorganized by ECM producing cells, degrading enzymes, and autophagy. Cells in the ECM produce new ECM for remodeling and repair. Chronic or severe tissue injuries can cause excessive ECM production, with increased ECM stiffness leading to fibrosis. ECM provides transparency to the cornea by the specific distribution of collagen fibrils and proteoglycans. ECM consists of a basement membrane and interstitial matrix. The basement membrane consists of collagen IV, laminins, perlecan, agrin, nidogen, and entactin. The interstitial matrix consists of collagens (collagen I), fibronectin, proteoglycans, glycosaminoglycans, tenascin C and elastin. The cornea is an avascular and highly innervated structure. The corneal ECM is regulated by several factors, including autophagy, wound healing process, myofibroblasts, TGF-β, PDGF, VEGF, FGF, MMPs/proteases and many other factors and cells, including limbus.

ECM forms a highly complex network of proteins and provides a supportive structure by interacting with various cells and/or factors (Figure 2). ECM is not a static system; rather, it is a dynamic structure which constantly remodels itself in tissues to maintain tissue-specific structures and functions in a given microenvironment [20]. The corneal stroma mainly consists of keratocytes/fibroblasts and collagen-rich ECM [54]. Keratocytes are the primary resident cell type, and predominantly maintain ECM levels in the tissue. The resident cells in the ECM secrete many substances that interact and regulate ECM components in the tissue microenvironment [18]. ECM consists of fibrous organizing proteins, including collagens, elastins, fibronectins, laminins, glycoproteins, proteoglycans, and glycosaminoglycans. Basement membranes contain collagen type IV, laminins, nidogen 1 and 2, perlecan, agrin, collagen type XV, and collagen type XVIII. The interstitial matrix consists of collagens (collagen I), fibronectin, proteoglycans (PGs), glycosaminoglycans (GAGs), tenascin C and elastin [15,20]. Several growth factors, cytokines and chemokines are deposited in the ECM by binding to specified ECM components; they are subsequently released when required [17]. Additionally, ECM holds water, hydrates tissues, and functions as a selective barrier to the external environment. 

A recent report indicated that proteoglycans and proteins of ECM components such as decorin, biglycan, endorepellin, endostatin, collagen VI, and plasminogen kringle 5 strongly induce autophagic mechanisms by receptors and downstream signaling pathways. In contrast, ECM components laminin α2, perlecan, and lumican inhibit autophagy [55]. Decorin is a small, leucine-rich proteoglycan with antifibrotic and anti-angiogenic properties in the cornea. It plays an essential role in the wound healing process and inhibits TGF-β1, 2, 3 with equal efficiency to reduce TGF-β-associated fibrosis [37,56,57,58]. Decorin upregulates the expression of Beclin-1, phosphorylates AMPK, stimulates paternally expressed gene 3 (Peg3), and activates autophagy by interacting with vascular endothelial growth factor (VEGF) receptor 2 (VEGFR2) in endothelial cells [55,59]. Moreover, decorin stimulates myostatin through Met-receptors and activates mitophagy in endothelial cells [55]. Another proteoglycan, Biglycan, converts LC3-II, recruits p62, and promotes autophagy flux through TLR4 and CD44 [55]. Currently, we are studying the role of ECM component decorin in the regulation of autophagy, wound-healing mechanisms, and pathologic signaling pathways in the cornea. Fragments of ECM can also induce autophagy.

ECM provides transparency for the cornea through the precise distribution of collagen fibrils and proteoglycans. Some 90% of the cornea is made up of about 200 collagen lamellae that crisscross it in various directions [60]. Corneal fibrosis is induced mainly by activated keratocytes/fibroblasts producing myofibroblasts, but sometimes by other cells in the stroma. TGF-β signaling is the most well-known and robust inducer of fibrosis in the cornea [20]. ECM in the cornea is made up of different types of collagens. Collagen fibril bundles form collagen fibers. Collagen IV is the main constituent in the BM. Collagen I, III and V are the most important types in the stroma, and are also present in the Bowman’s layer. Collagen VIII is present in the Descemet membrane. The presence or deposition of altered or different collagen types in these layers can affect the wound healing process [15]. 

## 4. Wound Healing

**Figure 3:**
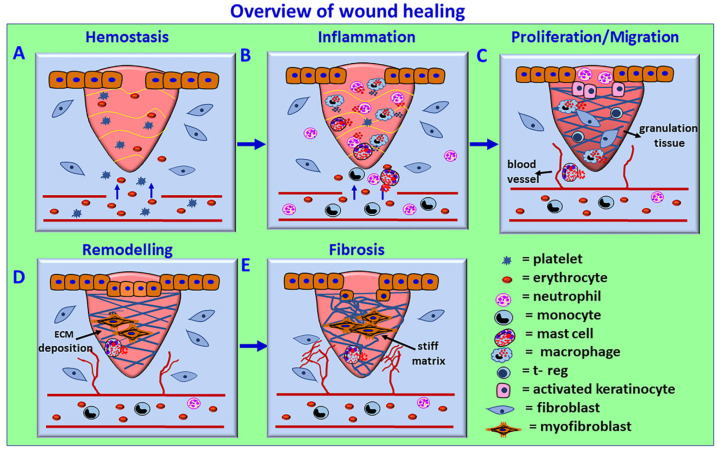
Schematic overview of the wound healing process. The process consists of four phases: (**A**) coagulation and hemostasis, (**B**) inflammation, (**C**) proliferation, and (**D**) wound remodeling and (**E**) scar tissue formation. In the first phase, blood clots (fibrin clots) form immediately after an injury and prevent further bleeding from the damaged blood vessels by vasoconstriction, hemostasis, and platelet plug formation. Activated platelets release cytokines and growth factors such as TGF-β and PDGF, initiate an immune response, and attract inflammatory neutrophils and monocyte and M1 inflammatory macrophages. The inflammation phase controls bleeding and prevents infections by removing damaged cells and pathogens from the wound area by neutrophils, followed by macrophages. Chemokines, DAMPs, and other factors from the injured cells also induce the recruitment of neutrophils to perform phagocytosis and NET generation to clean up the injury site and remove damaged ECM components. Macrophage-released mediators further attract immune cells for wound healing. Mast cell activation-derived cytokines, chemokines, and vasoactive mediators play an essential role in increased vasodilatation, vascular permeability, and inflammatory cell recruitment. In the proliferative phase, collagen and ECM rebuild new tissue (pink granulation tissue) with new blood vessels, and the wound contracts by myofibroblast contraction. Epithelial cells reappear on the wound surface (epithelization). In the wound remodeling phase, the collagens are reorganized, the wound is fully closed, and the cells that are no longer needed are removed by apoptosis. Defective or failure of the wound healing phases can cause chronic wounds. The persistence of myofibroblasts and excess and stiff ECM deposition can cause scar and fibrotic conditions. The avascular corneal wound healing occurs without blood vessel rupture in the cornea.

Platelets, neutrophils, monocytes/macrophages, lymphocytes, mast cells, and fibroblasts are the primary cell types implicated in the wound healing process [61]. Monocytes differentiate into macrophages and dendritic cells [2]. The wound healing mechanism involves four phases: (a) coagulation and hemostasis, (b) inflammation, (c) proliferation, and (d) wound remodeling and scar formation, as shown in Figure 3. 

In the first phase, blood clots (fibrin clots) form immediately after an injury and prevent further bleeding from the injured blood vessels by vasoconstriction, hemostasis, and platelet plug formation. Activated platelets release many cytokines and growth factors such as TGF-β and platelet-derived growth factor (PDGF), initiate an immune response and attract inflammatory neutrophils and monocyte and M1 (proinflammatory) macrophages. The inflammation phase controls bleeding and prevents infections by removing damaged cells and pathogens from the wound area by neutrophils, and then macrophages, through phagocytosis and the generation of ROS. Neutrophils also degranulate and release microbicidal agents. Chemokines, DAMPs and other factors from the injured cells induce the recruitment of frontline innate immune cell neutrophils through transendothelial migration to perform phagocytosis and NET generation, remove damaged ECM and clean up the injured site. The NETs released in the extracellular space trap the microbes/pathogens that enter after the tissue injury. NETs also promote the differentiation of fibroblasts into myofibroblasts, and are found close to the fibroblasts expressing α-SMA in fibrotic disease conditions [62]. Further, NETs are implicated in the resolution of inflammation by binding and sequestering inflammatory cytokines which are then degraded by the proteases that are attached to the NETs [62,63]. Neutrophils from blood vessels enter the intercellular space in the injured/infected region in the tissues through transendothelial migration by chemotaxis. Once their phagocytic and NET functions are complete, excessively recruited neutrophils return to the blood vessel from the site of injury/infection through reverse transendothelial migration as a round trip and prevent excessive tissue damage or any damage to the normal tissues at the site of injury/infection [64]. Neutrophils also contribute to ECM modulation and functions through different mechanisms, such as the release of matrix metalloproteinases, NET formation, etc. [65]. The role of NETs in corneal wound healing and homeostasis is not clearly known. However, previous studies reported that NETs were detected on the ocular surface in dry eye disease (DED) and other corneal disorders such as alkali burn, indicating that the presence of NETs in the precorneal and cornea may induce corneal inflammation and corneal neovascularization [66,67]. The mediators released from the activated macrophages attract more immune cells for the wound healing process. Mast cell activation-mediated release of cytokines, chemokines and growth factors, as well as vasoactive factors, also play a crucial role in inflammatory cell recruitment to the site of injury/infection. 

In the proliferative phase, collagen and ECM rebuild new tissue (pink granulation tissue) with new blood vessels, and the wound contracts by myofibroblast contraction. Epithelial cells appear on the wound surface (epithelization). In the wound remodeling phase, collagens are reorganized, the wound is fully closed, and the cells that are no longer needed are removed by apoptosis. Once the wound healing process is complete, myofibroblasts generally vanish by apoptosis or transdifferentiate again into keratocytes [68]. Activated myofibroblast produces ECM components such as collagen, glycosaminoglycans, tenascin-C, and fibronectin [18]. Several growth factors, such as TGF-β1, activins, connective tissue growth factor (CTGF), fibroblast growth factor (FGF), PDGF, epidermal growth factor (EGF), insulin-like growth factors (IGFs), VEGF, bone morphogenic protein (BMPs), and hepatocyte growth factor (HGF), control ECM deposition in tissue [18]. Undesirable, defective or failure of progression of the normal wound healing phases can cause chronic wounds. The persistence of myofibroblasts in the injured area with continuous production and excess deposition of ECM can cause scarring and fibrotic conditions. 

The ECM regulates wound healing by controlling the cells in the wound site to proliferate, migrate, and effect tissue formation in association with the autophagic process. Corneal wound healing is complicated and involves different processes, as there are no blood vessels in the cornea. The corneal wound healing process constitutes a resurfacing of corneal epithelial cells, a reorganization of the basement membrane, and the regeneration of ECM components [69]. The cellular immune response in the limbal precorneal region plays an essential role in these processes, maintaining the corneal epithelium and contributing to epithelial wound healing [70,71]. Corneal wound healing is discussed in a separate section below. 

## 5. Mast Cells in Wound Healing and Fibrosis

Mast cells are important effector cells of innate and acquired immune responses. They are distributed ubiquitously throughout the body [72,73,74]. Their activation leads to the release of pre-activated, prepackaged and newly-synthesized mediators such as histamine, proteases, anti- and pro- inflammatory cytokines/chemokines, pro-angiogenic factors and neurotrophic factors, and contributes to tissue protection, allergic reactions, protection against infections, and wound healing. Mast cells are present in the cornea, conjunctiva, limbus, choroid, iris, optic nerve and its meninges, as well as in limited numbers in the cornea. They release various multifunctional pro- and anti- inflammatory cytokines, chemokines, and growth factors [75,76,77,78,79]. Additionally, they are present in the corneoscleral, precorneal limbus region that is highly innervated and vascularized, and houses stem cells [80]. A large number of mast cells are present in the tissues that are directly exposed to the outer environment, such as skin, the respiratory tract, gastrointestinal tract etc. Mast cells are involved in the first line of defense against invading pathogens, trauma and adverse environmental conditions such as temperature, humidity, pressure, allergens and environmental toxins [81,82]. Mast cells are early responders in the immune system and interact with environmental antigens and toxins during the immune response by releasing prestored and preactivated mediators [74,83]. They are involved in many diseases and inflammatory conditions, including allergic conjunctivitis of the eye [84,85,86]. Topical eye drops with mast cell inhibitors and antihistamines are used to inhibit mast cell activation in the ocular surface in allergic conjunctivitis [87,88,89,90]. Mast cells participate in murine corneal morphogenesis during the development, limbal vasculogenesis and corneal innervation by providing several growth factors; their numbers decline after development [80]. A recent study indicated that more mast cells are present in the nasal limbus side of the cornea than the temporal side. Therefore, neovascularization is greater on the nasal side than the temporal side of injured cornea [91]. Mast cells initiate the recruitment of innate immune cells, including neutrophils, after ocular surface injury [92]. Mast cell activation in the ocular surface enhances corneal neovascularization [77]. 

An injured cornea can activate and degranulate nearby mast cells and release various pro- and anti-inflammatory mediators that may provide the initial protective effects; however, excessive and sustained mast cell activation can cause a harmful inflammatory response. Several multifunctional cytokines, chemokines, growth factors, and vasoactive factors including IL-1β, IL-6, IL-8, TNF-α, IL-13, TGF-β1, VEGF, PDGF, FGF-2, keratinocyte growth factor, histamine, proteases (tryptase & chymase) and chemokine (C-C motif) ligand 2 (CCL2)/monocyte chemoattractant protein 1 (MCP1) that are released from activated mast cells play an important role in many phases of the wound healing process, angiogenesis, collagen production and ECM modulation, and tissue repair [93,94]. 

Mast cells are implicated in all phases of wound healing (hemostasis, inflammation, proliferation, remodeling) and also in fibrosis/scar/hypertrophic scar formation by releasing multifunctional preformed, preactivated and de novo synthesized mediators. Briefly, mast cells contribute to hemostasis/clot formation and clot stabilization through factor XIIIa and TNF-α. CCL2, i.e., stem cell factor (SCF) from keratinocytes/macrophages, recruits mast cells to the site of injury. Vasoactive inflammatory mediators such as histamine, VEGF, IL-6, chemokine (C-X-C motif) ligand 2 (CXCL2), and IL-8 released from mast cells augment endothelial permeability and vasodilation, and recruit neutrophils and monocytes to the site of injury in the inflammatory phase. Mast cell tryptase acts on endothelial cells through protease-activated receptor 2 (PAR-2), causes vasodilatation and facilitates neutrophil and other inflammatory cell migration to the site of injury. Recruited neutrophils produce IL-1α and TNF-α, and activate local fibroblasts and keratinocytes in the inflammatory stage. Mast cells released mediators such as IL-4, VEGF, and bFGF are implicated in the activation, proliferation and migration of keratinocytes, endothelium and fibroblasts, as well as in the reepithelialization process and the angiogenesis which occurs in the proliferative phase of wound healing [93,95]. Mast cells activate fibroblasts to proliferate and produce new ECM through the release of L-4, VEGF, and bFGF. TGF-β can regulate/suppress immune cells, including mast cells, in an autocrine and paracrine fashion [94]. In the remodeling phase or during scar formation, mast cell-derived mediators induce myofibroblast differentiation, smooth muscle alpha-actin (α-SMA) formation, collagen production, and scar formation by secreting proteases that cleave ECM. Mast cell-derived FGF-2, VEGF, PDGF, TGF-β, nerve growth factor (NGF), IL-4, and IL-8 enhance neoangiogenesis, fibrinogenesis, and reepithelialization in the tissue repair process. 

Mast cell-derived TGF-β1 is a profibrotic and growth factor that augments fibroblast proliferation and matrix production. Mast cell-derived TGF-β2 increases collagen deposition in the ECM [74]. Mast cells promote angiogenesis to nourish newly produced cells, and can also directly interact with fibroblasts and cause fibrosis by releasing TGF-β and chymase and through the formation of angiotensin II [93]. Previous studies have shown mast cell numbers and their activation is reduced in tissues that heal with fewer fibrotic scars. However, increased mast cell number and activation are associated with edema, scar/fibrosis, and chronic allograft rejections [96,97]. Mast cells can limit inflammation by releasing anti-inflammatory molecules to enhance wound healing [93,95]. Mast cell-derived proteases tryptase and chymase degrade ECM and facilitate tissue angiogenesis [98,99]. Chymase can degrade the basement membrane [100]. Mast cell-derived histamine and tryptase are crucial for the proliferation of fibroblasts, collagen production by fibroblasts, collagen deposition in the ECM, α SMA contraction, and wound contraction [99]. 

Activated platelets in the inflammation phase of wound healing express autophagy markers Beclin-1, LC3 and ATG7, indicating that the autophagy process is underway in the platelets [28]. Resting platelets show a basal level of autophagy, the disruption of which is characterized by decreased platelet activity after an injury and hemostasis. Autophagy is involved in platelet activation-associated signals to the tissue surrounding the wounded area to recruit immunocytes such as neutrophils and M1 macrophage for the removal of pathogens, damaged cells, ECM and debris [28]. 

## 6. Corneal Wound Healing and Repair

**Figure 4:**
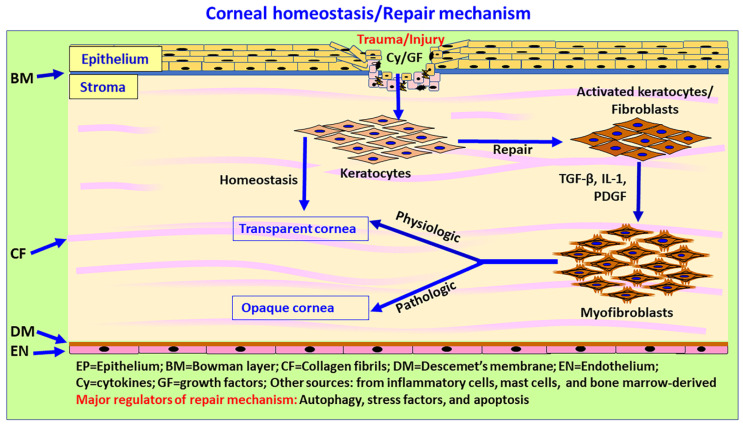
Schematic diagram showing autophagy, ECM, wound healing, and scar and fibrous tissue formation in the cornea. The cornea consists mainly of epithelium, stroma and endothelium. ECM components such as decorin, collagen VI, laminin alpha-2, endostatin, endorepellin and krigle V modulate autophagy in the cornea. Corneal wound healing is a complex mechanism due to the lack of blood vessels in normal cornea. During corneal wound healing, keratocytes undergo apoptosis or necrosis, while the quiescent keratocytes in the stroma become activated by cytokines, proliferate, migrate and transform into fibroblasts and contractile, opaque myofibroblasts (repair phenotypes) through TGF-β signaling and the secretion of ECM components in the injury site. Myofibroblasts migrate to the wound site and release ECM components such as collagen types I, III, IV and V to induce wound healing. Inflammatory mediators released from the epithelium and stromal cells chemoattract immune/inflammatory cells to clear the cellular debris, dead cells, and damaged ECM. An altered ratio of collagen I/III leads to corneal opacity after corneal injury. Once the wound healing process is complete, myofibroblasts generally disappear by apoptosis or transdifferentiate into keratocytes, and the stroma restores a normal structure and function, albeit with or without normal stromal transparency. Limbal stem cells, as well as immune and inflammatory cells, could contribute to immune-privileged corneal wound healing and homeostasis. The process of scleral, precorneal and limbal immune and inflammatory cell infiltration, including mast cells in autophagy, ECM modulation and corneal wound healing, is not yet clearly understood.

An injured or opaque cornea causes blindness. The completion of the corneal wound healing process results in transparency and normal vision. Despite significant progress in recent years, the corneal wound healing mechanism remains poorly understood (Figure 4) [101]. Corneal transparency and avascularity is important for normal vision, but is affected by inflammation, fibrosis, neovascularization, and limbal defect. Thus, corneal immune privilege is essential for corneal transparency. However, a limited immune response is important for normal wound healing and for the restoration of a normal cornea [60]. This process involves limbal stem cells, apoptosis, necrosis, migration, proliferation, differentiation of corneal cells, and ECM remodeling [38]. The human cornea is made up of six layers, namely, epithelial cells, basement membrane, Bowman’s layer, stroma, Descemet membrane, and corneal endothelial cells. The stroma is the thickest part, constituting about 85% of the cornea. The stroma consists of type I and V collagen fibers, specifically arranged to provide transparency to the cornea for normal vision. The presence of a basal level of autophagy is essential for corneal homeostasis and defense against invading pathogens, as well as against adverse environmental conditions. Autophagy is implicated in corneal cells and disorders such as epithelium, endothelium, keratoconus, dry eye disease, corneal wound healing and fibrosis, corneal haze, and infections [9]. 

In mild corneal injury, limited keratocyte apoptosis occurs. Subsequently, the epithelium regenerates, the epithelial basement membrane and Descemet’s basement membranes are repaired, and keratocyte or fibrocyte-derived myofibroblast precursors either undergo apoptosis or are converted back to parent cell types [102]. However, in severe injuries, profibrotic TGF-β and PDGF induce the formation of αSMA and myofibroblasts that secrete excess ECM components and produce stromal fibrosis scarring [102]. In normal conditions, i.e., without any corneal injury, epithelial-derived TGF-β and PDGF production are low, and basement membrane (EBM) and Descemet’s basement membrane (DBM) prevent these factors from entering the stroma. However, following injury to the EBM or DBM, both TGF-β and PDGF, along with other factors, enter the stroma in high quantities and activate uninjured live keratocytes, transforming them into fibroblasts. These fibroblasts and corneal fibrocytes differentiate into opaque, motile/contractile mature myofibroblasts [102,103]. 

The damaged corneal epithelium undergoes apoptosis or necrosis within a few minutes, and releases IL-1 that enters the stroma. The IL-1 and TNF-α released from the epithelium induce apoptosis of keratocytes [15]. IL-1 activated keratocytes and fibroblasts, along with damaged keratocytes, secrete many cytokines, chemokines, and growth factors that chemoattract many immune/inflammatory cells (monocytes, macrophages, lymphocytes, fibrocytes, and other cells) from bone marrow to the injured site in the stroma. IL-1 also induces the release of keratinocyte growth factor (KGF) and hepatocyte growth factor (HGF) from the keratocytes and fibroblasts and regulates the proliferation, migration, and differentiation of healing epithelial cells [102]. IL-1 from damaged epithelial cells also upregulates the synthesis and release of metalloproteinases and collagenases from keratocytes, fibroblasts and myofibroblasts, and reorganizes the stromal ECM after injury (Figure 4). Along with IL-1, TGF-β1 and PDGF are also released from the corneal epithelial and endothelial cells, subsequently entering the stroma and restoring its normal structure in months or years. EBM and DBM which regulate TGF-β and PDGF entry into stroma include collagen IV and perlecan for collagen and nidogens for PDGF. Injury to the epithelium and EBM or endothelium-DBM causes TGF-β and PDGF to enter the stroma and activate surviving keratocytes to form fibroblasts; these fibroblasts and corneal fibrocytes mature into myofibroblasts. Insufficient levels of TGF-β and PDGF can induce apoptosis in these cells. 

Mild corneal injuries usually heal without differentiation into mature myofibroblasts or scarring. However, in severe corneal injury, the regeneration of EBM or DBM may take months to years, or may never reach completion. In these conditions, many mature myofibroblasts remain, causing stromal fibrosis. Myofibroblasts are opaque and produce large amounts of disordered ECM that cause corneal fibrosis [102]. If the EBM and DBM are regenerated, TGFβ and PDGF levels decrease, myofibroblasts undergo apoptosis, and keratocytes repopulate the affected stromal region. These keratocytes remove disordered ECM, restore normal stroma, and remove corneal opacity in months to years, although in some cases, this does not happen, resulting in permanent corneal scarring (fibrosis). Epithelial cells and keratocytes synthesize perlecan, nidogen-1, nidogen-2, laminins, and other ECM components. Restoring tissue integrity requires the production and deposition of ECM in the affected regions. Immediately after reepithelization and completion of the epithelium barrier, stromal immune cells remove the debris of immune cells and collagen from the stroma [49].

The corneal wound healing process starts immediately after injury, through the release cytokines, chemokines and other mediators from epithelium, keratocytes and fibroblasts, which chemoattract various immune/inflammatory cells (monocytes, macrophages, lymphocytes, and other cells) from bone marrow to the injured site in the stroma. These cells also secret many of these mediators and amplify the response. Keratocytes transform into mature myofibroblasts. If the injured EBM regenerates and blocks the entry of IL-1, TGF-β, PDGF, and other cytokines and chemokines, corneal homeostasis is restored within days. However, if the EBM fails to regenerate, then fibrosis develops [102]. The cornea is highly innervated, and thus, corneal damage also damages nerves, and the process of reinnervation takes months to complete. Additionally, myofibroblasts and the fibrosis process can inhibit the innervation process.

Wound healing generally requires angiogenesis. However, the cornea has no blood vessels or lymph vessels, and most mild stromal wounds heal without forming new blood vessels or corneal neovascularization [15]. However, in severe stromal injury, blood vessels and lymph vessels develop during the healing process. These new blood vessels can develop from endothelial cells in the corneal limbus and cells from bone marrow. Blood vessels grow in the presence of angiogenic factors such as VEGF, TGF-β, PDGF, IL-1 and bFGF in the stroma released from corneal epithelial and endothelial cells, stromal cells, and immune/inflammatory cells. VEGF is a crucial cytokine in the neovascularization process. It promotes endothelial cell proliferation and increases vascular permeability. Beclin-1 is a crucial protein in autophagy, and the Beclin-1 shRNA (short hairpin ribonucleic acid) can block VEGF and stop the neovascularization process [104]. Neovascularization in the cornea is regulated by the balance between proangiogenic factors and anti-antigenic factors such as endostatin, angiostatin, arrestin, restin, metalloproteinase 3, and other factors in the cornea [15]. Neovascularization can occur within three days after injury, peaking on the seventh day, but may also start to regress from the fourth day under the influence of VEGF implanted in the corneal micropocket in rabbit eyes [105]. The persistence of blood vessels can cause edema and may impair vision.

Autophagy may play a crucial role in corneal pathophysiological conditions by removing unwanted or dysfunctional intracellular components and supporting cell survival through the recycling of intracellular substances and organelles. Specific autophagy marker dynamics are implicated in corneal cells in the corneal wound healing process. Autophagy can also help in restoring normal ECM after corneal injury. Autophagy regulate all phases of the wound healing process [28]. Chronic wounds are continuously exposed to cellular debris, high levels of inflammatory cytokines/chemokines, and ischemic conditions, leading to higher cellular stress and the activation of autophagy to limit cellular stress. However, a continuously high level of stress leads to the apoptosis of cells and the failure to replace the affected tissue, resulting in a chronic wound. In scarring and fibrosis, the wound fails to resolve, with limited autophagy associated with apoptosis of more endothelium and elevated myofibroblasts produced ECM [28]. Corneal stromal wound healing takes place in four phases. In the first phase, the keratocytes at the injury site undergo apoptosis. In the second phase, adjacent keratocytes proliferate and differentiate into fibroblasts, and move into the wound area. In the third phase, fibroblasts are converted into myofibroblasts. In corneal stromal healing, keratocytes are transformed into contractile myofibroblasts by TGF-β [38]. In the fourth phase, remodeling of the stroma takes place, which may take about a year. Corneal endothelial dystrophies have elevated levels of autophagic proteins [10]. Matrix metalloproteinases (MMPs) are enzymes implicated in the degradation/destruction of ECM components and basement membrane, angiogenesis, and wound healing. MMP-14 is an important MMP associated with angiogenesis and ECM remodeling in the corneal homeostasis [27]. A previous study showed that corneal injury-associated mast cell activation significantly increased the levels of CCL2 and TNF-α and neutrophil recruitment [92]. 

## 7. Dysregulated or Delayed Autophagy and Its Late Effects

Corneal injuries are common among active military personnel, veterans, and civilians impacted by terrorist attacks due to exposure to toxic gas, combat blasts, blast waves, and TBI [106]. TBI induces invisible injury in the brain, glial activation, neuroinflammation, neurodegeneration, dementia, vascular/BBB disruption and ocular disturbances, and is a risk factor for the pathogenesis of post-traumatic stress disorder (PTSD) and chronic neurodegenerative diseases including Alzheimer’s disease in the late life [107,108,109,110]. Concussion/mild TBI-induced BBB disruption/pathogenesis may be acute and resolved within hours to days. TBI-induced pathogenesis can induce short and late/delayed biphasic effects. The delayed or late effects of TBI may last for weeks, months, years and even decades after the initial TBI event [107,111]. Blast-associated head injuries may be due to multiple causes such as blast waves, flying objects, exposure to toxic gas, thermal burns, or radiation exposure. TBI/combat blast exposure can cause several visual impairments, closed eye injuries and ocular surface/corneal disorders such as DED, corneal stromal scars, Descemet membrane damage, reduced endothelial cells, foreign bodies in corneal and conjunctival tissues, and retinal damage [112,113,114,115]. Likewise, warfare and toxic chemicals such as hydrogen sulfide, mustard gas, carbofuran, acrolein, chlorine, etc. cause several corneal abnormalities [31,33,116,117]. Various ocular pathologies such as ocular edema, redness, tearing, itching, abrasion, corrosions, corneal injury, neovascularization, blurred vision, and loss of vision in humans and animals have been observed following exposure to industrial chemicals (alkali, acids, anhydrous ammonia, phosphine), warfare agents (mustard gas, chlorine, acrolein, phosgene oxime, lewisite) and pesticides (carbofuran, ethylene oxide) [32,33,35,118,119,120,121,122,123,124]. Our recent studies and research reported in the literature have led us to postulate that irregular autophagosomal and lysosomal biogenesis leads to improper clearance of injurious factors and the accumulation of unwanted and harmful molecules/proteins in keratocytes, ultimately resulting in corneal pathologies. 

**Figure 5:**
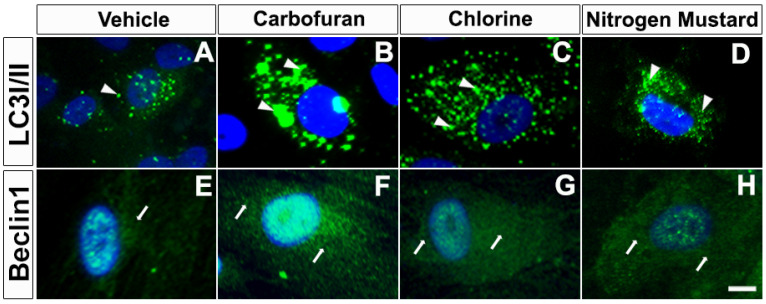
Carbofuran, chlorine, and nitrogen mustard gas upregulate the expression of autophagy in primary human corneal fibroblasts (hCSF). Immunofluorescence showing LC3I/II (**A**–**D**) and Beclin1 (**E**–**H**) expression. hCSFs which had been exposed to carbofuran (10 μM), chlorine (0.001% NaOCI) or nitrogen mustard gas (200 µM) showed markedly increased levels of Beclin 1 and LC3I/II as compared to vehicle controls. Scale bar = 25 µM.

**Figure 6:**
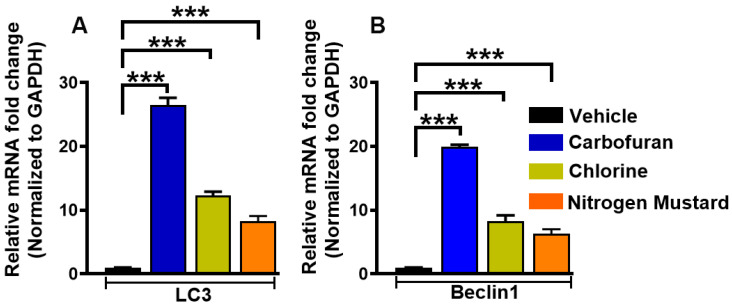
Carbofuran, chlorine, and nitrogen mustard gas upregulate the expression of LC3 and Beclin 1 mRNA in primary human corneal fibroblasts (hCSF). hCSFs which had been exposed to carbofuran (10 μM), chlorine (0.001% NaOCI) or nitrogen mustard gas (200 µM) showed significantly increased expression of LC3 (**A**) and Beclin 1 (**B**) mRNA as compared to vehicle-treated controls (*** *p* < 0.001).

**Figure 7:**
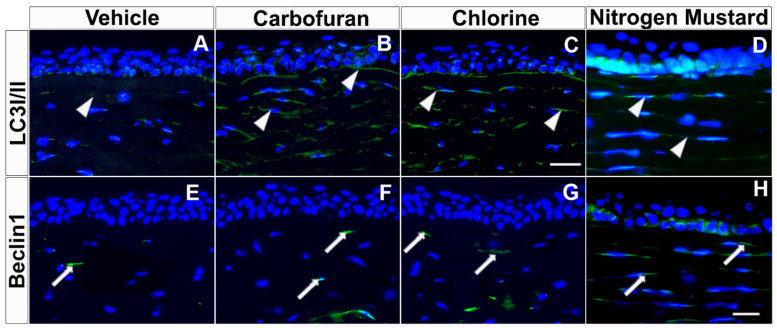
Carbofuran, chlorine, and nitrogen mustard gas upregulate the expression of LC3I/II and Beclin 1 in ex vivo human cornea organ culture model. Immunofluorescence showing LC3I/II (**A**–**D**) and Beclin1 (**E**–**H**) expression. Donor human corneas exposed to carbofuran (10 μM), chlorine (0.001% NaOCI) or nitrogen mustard gas (200 µM) showed markedly increased levels of Beclin 1 and LC3I/II as compared to vehicle controls. Scale bar = 50 µm.

Autophagy-related genes (ATGs) are constitutively expressed at high levels in the cornea, lens, retina, and other eye tissues [13]. Corneal stroma contain numerous autophagic markers, including LC3/Atg8, BECN1/Atg6, Atg5, DRAM1, SQSTM1/p62 and PRKN2 [9]. The inhibition of autophagy can inhibit the formation of neovascularization induced by VEGF [104]. Dysfunctional autophagy is implicated in corneal diseases such as granular corneal dystrophy type 2, keratoconus, dry eye, and Schnyder corneal dystrophy [9,125,126,127]. Keratocytes in the stroma play a significant role in corneal repair by producing myofibroblasts through several cytokines, growth factors, and extracellular matrix (ECM) components. A clear cornea lacks myofibroblasts, but these are formed in stroma from keratocytes after injury for wound healing by synthesizing and secreting ECM components, collagens, and α-smooth muscle actin (α-SMA) stress fibers. Once the cornea has healed, myofibroblasts and excessive ECM proteins typically degrade via autophagy and/or apoptotic pathways. Our recent studies with normal and keratoconus donor human cornea-derived primary culture and organ culture models characterized the expression of autophagy markers in corneal epithelial, stromal fibroblasts, and endothelial cells [9]. Furthermore, human corneal fibroblast primary cultures and organ cultures exposed to chemical threats revealed the involvement of autophagic genes/mechanisms. In these experimental in vitro models, exposure to carbofuran, chlorine or nitrogen mustard gas increased the expression of autophagy signature genes Beclin 1 and LC3I/II mRNA as shown in hCSF (Figure 5 and Figure 6) ex vivo human cornea organ culture model (Figure 7).

LC3 is an essential protein in the formation of the autophagosome. It is converted from its cytosolic form (LC3I) to an active membrane-bound form (LC3 II) during the formation of the autophagosome. Beclin 1 initiates autophagosome (phagophore) formation [9]. 

In conclusion, the results of this pilot study indicate that a common autophagy mechanism is implicated in carbofuran-, chlorine-, and nitrogen mustard gas-induced corneal pathologies; however, the exact mechanisms for this are not yet known, although work is ongoing to shed further light on this matter in our laboratory. Chemical threat agent/toxin-induced corneal injury may be repaired within days/weeks, or may result in chronic delayed corneal damage due to defective autophagy, as seen in Keratoconus disease [9,128,129]. Autophagy may mediate antioxidative stress, anti-apoptosis and anti-inflammatory effects in TBI. Changes in the autophagic markers or autophagy associated with increased levels of Beclin 1, p62, LC3 after TBI protect neurons from neurodegeneration [130,131]. Autophagic clearance has been found to be impaired soon after TBI, leading to neuronal death [132,133,134]. Thus, both TBI and specific toxic agents can have acute effects, as well as delayed chronic effects, after the initial TBI incident. These delayed effects may be due to the dysregulated or delayed autophagy associated with the reduced clearance of waste substances, dysregulated/abnormal ECM production and deposition, and abnormally prolonged wound healing and scar formation; however, the exact mechanism for this is not yet known. 

## 8. Conclusions

Autophagy is a protective mechanism that recycles harmful cellular materials and debris, including nutrients, removes dysfunctional and damaged cell organelles, and controls immune and inflammatory cells to regulate cellular homeostasis. Thus, efficient autophagy is required to maintain cellular homeostasis, and its dysregulation leads to many pathological outcomes. Wound healing is a highly coordinated, complex process involving multiple cell types and their mediators. The wound healing process efficiently repairs injured tissue, allowing it to regain its normal structure and function. Autophagy, ECM, and wound healing processes are intertwined and regulate the homeostasis of cells, tissues and organs. Dysregulation and delayed autophagy, ECM, and wound healing lead to many corneal pathologies and disorders. Autophagy in neutrophils, monocytes and macrophages modulates their phagocytic functions and the release of cytokines/chemokines. Mast cells, which play an essential role in the immune and inflammatory response, ECM modulation and wound healing, express autophagy markers that are involved in the degranulation and release of mediators. However, the contribution that mast cells and other immunocytes make to autophagy, ECM organization and corneal wound healing mechanisms is not yet clearly understood. ECM is a dynamic structure which is constantly remodeled in all tissues to maintain proper structure and functions. ECM components also regulate autophagy in a cell- and tissue-/organ- specific manner. The cornea expresses various autophagic markers that play an essential role in corneal homeostasis. The ECM in the cornea is a highly organized structure which provides corneal transparency for normal vision. The cornea is avascular, and ECM and wound healing processes are unique, in the sense that they are regulated by autophagy to prevent immune cell infiltration, neovascularization, fibrosis, and scar formation in corneal disorders, due to excessive immune response after corneal injury or infection. 

## Data Availability

The data are available with the corresponding author and will be provided upon reasonable request.

## References

[1] Chang N.C. (2020). Autophagy and Stem Cells: Self-Eating for Self-Renewal. Front. Cell Dev. Biol..

[2] Germic N., Frangez Z., Yousefi S., Simon H.U. (2019). Regulation of the innate immune system by autophagy: Monocytes, macrophages, dendritic cells and antigen presentation. Cell Death Differ..

[3] Parzych K.R., Klionsky D.J. (2014). An overview of autophagy: Morphology, mechanism, and regulation. Antioxid. Redox Signal..

[4] Wang L., Das J.K., Kumar A., Peng H.Y., Ren Y., Xiong X., Yang J.M., Song J. (2021). Autophagy in T-cell differentiation, survival and memory. Immunol. Cell Biol..

[5] Deretic V. (2021). Autophagy in inflammation, infection, and immunometabolism. Immunity.

[6] Belgrad J., De Pace R., Fields R.D. (2020). Autophagy in Myelinating Glia. J. Neurosci..

[7] Fernandez-Albarral J.A., de Julian-Lopez E., Soler-Dominguez C., de Hoz R., Lopez-Cuenca I., Salobrar-Garcia E., Ramirez J.M., Pinazo-Duran M.D., Salazar J.J., Ramirez A.I. (2021). The Role of Autophagy in Eye Diseases. Life.

[8] Ma S., Yu Z., Feng S., Chen H., Chen H., Lu X. (2019). Corneal autophagy and ocular surface inflammation: A new perspective in dry eye. Exp. Eye Res..

[9] Martin L.M., Jeyabalan N., Tripathi R., Panigrahi T., Johnson P.J., Ghosh A., Mohan R.R. (2019). Autophagy in corneal health and disease: A concise review. Ocul. Surf..

[10] Frost L.S., Mitchell C.H., Boesze-Battaglia K. (2014). Autophagy in the eye: Implications for ocular cell health. Exp. Eye Res..

[11] Klionsky D.J., Petroni G., Amaravadi R.K., Baehrecke E.H., Ballabio A., Boya P., Bravo-San Pedro J.M., Cadwell K., Cecconi F., Choi A.M.K. (2021). Autophagy in major human diseases. EMBO J..

[12] Levine B., Mizushima N., Virgin H.W. (2011). Autophagy in immunity and inflammation. Nature.

[13] Chai P., Ni H., Zhang H., Fan X. (2016). The Evolving Functions of Autophagy in Ocular Health: A Double-edged Sword. Int. J. Biol. Sci..

[14] Boya P., Esteban-Martinez L., Serrano-Puebla A., Gomez-Sintes R., Villarejo-Zori B. (2016). Autophagy in the eye: Development, degeneration, and aging. Prog. Retin. Eye Res..

[15] Kamil S., Mohan R.R. (2021). Corneal stromal wound healing: Major regulators and therapeutic targets. Ocul. Surf..

[16] Netto M.V., Mohan R.R., Ambrosio R., Hutcheon A.E., Zieske J.D., Wilson S.E. (2005). Wound healing in the cornea: A review of refractive surgery complications and new prospects for therapy. Cornea.

[17] Theocharis A.D., Skandalis S.S., Gialeli C., Karamanos N.K. (2016). Extracellular matrix structure. Adv. Drug Deliv. Rev..

[18] Pompili S., Latella G., Gaudio E., Sferra R., Vetuschi A. (2021). The Charming World of the Extracellular Matrix: A Dynamic and Protective Network of the Intestinal Wall. Front. Med..

[19] Frantz C., Stewart K.M., Weaver V.M. (2010). The extracellular matrix at a glance. J. Cell Sci..

[20] Bonnans C., Chou J., Werb Z. (2014). Remodelling the extracellular matrix in development and disease. Nat. Rev. Mol. Cell Biol..

[21] Chaurasia S.S., Lim R.R., Lakshminarayanan R., Mohan R.R. (2015). Nanomedicine approaches for corneal diseases. J. Funct. Biomater..

[22] Mohan R.R., Martin L.M., Sinha N.R. (2021). Novel insights into gene therapy in the cornea. Exp. Eye Res..

[23] Mohan R.R., Sharma A., Netto M.V., Sinha S., Wilson S.E. (2005). Gene therapy in the cornea. Prog. Retin. Eye Res..

[24] Mohan R.R., Rodier J.T., Sharma A. (2013). Corneal gene therapy: Basic science and translational perspective. Ocul. Surf..

[25] Espana E.M., Birk D.E. (2020). Composition, structure and function of the corneal stroma. Exp. Eye Res..

[26] Chen S., Mienaltowski M.J., Birk D.E. (2015). Regulation of corneal stroma extracellular matrix assembly. Exp. Eye Res..

[27] Pouw A.E., Greiner M.A., Coussa R.G., Jiao C., Han I.C., Skeie J.M., Fingert J.H., Mullins R.F., Sohn E.H. (2021). Cell-Matrix Interactions in the Eye: From Cornea to Choroid. Cells.

[28] Sylakowski K., Wells A. (2021). ECM-regulation of autophagy: The yin and the yang of autophagy during wound healing. Matrix Biol..

[29] Almadani Y.H., Vorstenbosch J., Davison P.G., Murphy A.M. (2021). Wound Healing: A Comprehensive Review. Semin. Plast. Surg..

[30] Flanagan G., Velez T., Gu W., Singman E. (2020). The Relationship between Severe Visual Acuity Loss, Traumatic Brain Injuries, and Ocular Injuries in American Service Members from 2001 to 2015. Mil. Med..

[31] Balne P.K., Sinha N.R., Hofmann A.C., Martin L.M., Mohan R.R. (2020). Characterization of hydrogen sulfide toxicity to human corneal stromal fibroblasts. Ann. N. Y. Acad. Sci..

[32] Gupta S., Fink M.K., Martin L.M., Sinha P.R., Rodier J.T., Sinha N.R., Hesemann N.P., Chaurasia S.S., Mohan R.R. (2020). A rabbit model for evaluating ocular damage from acrolein toxicity in vivo. Ann. N. Y. Acad. Sci..

[33] Fuchs A., Giuliano E.A., Sinha N.R., Mohan R.R. (2021). Ocular toxicity of mustard gas: A concise review. Toxicol. Lett..

[34] Rasiah P.K., Geier B., Jha K.A., Gangaraju R. (2021). Visual deficits after traumatic brain injury. Histol. Histopathol..

[35] Tripathi R., Balne P.K., Sinha N.R., Martin L.M., Kamil S., Landreneau J.R., Gupta S., Rodier J.T., Sinha P.R., Hesemann N.P. (2020). A Novel Topical Ophthalmic Formulation to Mitigate Acute Mustard Gas Keratopathy in vivo: A Pilot Study. Transl. Vis. Sci. Technol..

[36] McNutt P.M., Mohan R.R. (2020). The Need for Improved Therapeutic Approaches to Protect the Cornea against Chemotoxic Injuries. Transl. Vis. Sci. Technol..

[37] Mohan R.R., Tovey J.C., Gupta R., Sharma A., Tandon A. (2011). Decorin biology, expression, function and therapy in the cornea. Curr. Mol. Med..

[38] Ljubimov A.V., Saghizadeh M. (2015). Progress in corneal wound healing. Prog. Retin. Eye Res..

[39] De Munck D.G., De Meyer G.R., Martinet W. (2020). Autophagy as an emerging therapeutic target for age-related vascular pathologies. Expert Opin. Ther. Targets.

[40] Wu J., Lipinski M.M. (2019). Autophagy in Neurotrauma: Good, Bad, or Dysregulated. Cells.

[41] Doherty J., Baehrecke E.H. (2018). Life, death and autophagy. Nat. Cell Biol..

[42] Peng H., Park J.K., Lavker R.M. (2017). Autophagy and Macropinocytosis: Keeping an Eye on the Corneal/Limbal Epithelia. Investig. Ophthalmol. Vis. Sci..

[43] Santeford A., Wiley L.A., Park S., Bamba S., Nakamura R., Gdoura A., Ferguson T.A., Rao P.K., Guan J.L., Saitoh T. (2016). Impaired autophagy in macrophages promotes inflammatory eye disease. Autophagy.

[44] Ferguson T.A., Laurie G.W. (2016). Introduction to Autophagy in the Eye (or “What’s Eatin’ You?”). Exp. Eye Res..

[45] Ushio H., Ueno T., Kojima Y., Komatsu M., Tanaka S., Yamamoto A., Ichimura Y., Ezaki J., Nishida K., Komazawa-Sakon S. (2011). Crucial role for autophagy in degranulation of mast cells. J. Allergy Clin. Immunol..

[46] Nakano H., Ushio H. (2011). An unexpected role for autophagy in degranulation of mast cells. Autophagy.

[47] Rozman S., Yousefi S., Oberson K., Kaufmann T., Benarafa C., Simon H.U. (2015). The generation of neutrophils in the bone marrow is controlled by autophagy. Cell Death Differ..

[48] Botbol Y., Guerrero-Ros I., Macian F. (2016). Key roles of autophagy in regulating T-cell function. Eur. J. Immunol..

[49] Stepp M.A., Menko A.S. (2021). Immune responses to injury and their links to eye disease. Transl. Res..

[50] Liu Z., Chen D., Chen X., Bian F., Gao N., Li J., Pflugfelder S.C., Li D.Q. (2020). Autophagy Activation Protects Ocular Surface from Inflammation in a Dry Eye Model in vitro. Int. J. Mol. Sci..

[51] Wu T.T., Li W.M., Yao Y.M. (2016). Interactions between Autophagy and Inhibitory Cytokines. Int. J. Biol. Sci..

[52] Jang Y.J., Kim J.H., Byun S. (2019). Modulation of Autophagy for Controlling Immunity. Cells.

[53] Portillo J.A., Okenka G., Reed E., Subauste A., Van Grol J., Gentil K., Komatsu M., Tanaka K., Landreth G., Levine B. (2010). The CD40-autophagy pathway is needed for host protection despite IFN-Gamma-dependent immunity and CD40 induces autophagy via control of P21 levels. PLoS ONE.

[54] Sridhar M.S. (2018). Anatomy of cornea and ocular surface. Indian J. Ophthalmol..

[55] Schaefer L., Dikic I. (2021). Autophagy: Instructions from the extracellular matrix. Matrix Biol..

[56] Mohan R.R., Tandon A., Sharma A., Cowden J.W., Tovey J.C. (2011). Significant inhibition of corneal scarring in vivo with tissue-selective, targeted AAV5 decorin gene therapy. Investig. Ophthalmol. Vis. Sci..

[57] Balne P.K., Gupta S., Zhang J., Bristow D., Faubion M., Heil S.D., Sinha P.R., Green S.L., Iozzo R.V., Mohan R.R. (2021). The functional role of decorin in corneal neovascularization in vivo. Exp. Eye Res..

[58] Mohan R.R., Tripathi R., Sharma A., Sinha P.R., Giuliano E.A., Hesemann N.P., Chaurasia S.S. (2019). Decorin antagonizes corneal fibroblast migration via caveolae-mediated endocytosis of epidermal growth factor receptor. Exp. Eye Res..

[59] Buraschi S., Neill T., Goyal A., Poluzzi C., Smythies J., Owens R.T., Schaefer L., Torres A., Iozzo R.V. (2013). Decorin causes autophagy in endothelial cells via Peg3. Proc. Natl. Acad. Sci. USA.

[60] Mobaraki M., Abbasi R., Omidian Vandchali S., Ghaffari M., Moztarzadeh F., Mozafari M. (2019). Corneal Repair and Regeneration: Current Concepts and Future Directions. Front. Bioeng. Biotechnol..

[61] Shah J.M., Omar E., Pai D.R., Sood S. (2012). Cellular events and biomarkers of wound healing. Indian J. Plast. Surg..

[62] Sabbatini M., Magnelli V., Reno F. (2021). NETosis in Wound Healing: When Enough Is Enough. Cells.

[63] De Oliveira S., Rosowski E.E., Huttenlocher A. (2016). Neutrophil migration in infection and wound repair: Going forward in reverse. Nat. Rev. Immunol..

[64] Hirano Y., Aziz M., Wang P. (2016). Role of reverse transendothelial migration of neutrophils in inflammation. Biol. Chem..

[65] Zhu Y., Huang Y., Ji Q., Fu S., Gu J., Tai N., Wang X. (2021). Interplay between Extracellular Matrix and Neutrophils in Diseases. J. Immunol. Res..

[66] Reyes J.L., Vannan D.T., Eksteen B., Avelar I.J., Rodriguez T., Gonzalez M.I., Mendoza A.V. (2018). Innate and Adaptive Cell Populations Driving Inflammation in Dry Eye Disease. Mediat. Inflamm..

[67] Yuan K., Zheng J., Huang X., Zhang Y., Han Y., Hu R., Jin X. (2020). Neutrophil extracellular traps promote corneal neovascularization-induced by alkali burn. Int. Immunopharmacol..

[68] Lorenzo-Martin E., Gallego-Munoz P., Mar S., Fernandez I., Cidad P., Martinez-Garcia M.C. (2019). Dynamic changes of the extracellular matrix during corneal wound healing. Exp. Eye Res..

[69] Stuard W.L., Titone R., Robertson D.M. (2020). The IGF/Insulin-IGFBP Axis in Corneal Development, Wound Healing, and Disease. Front. Endocrinol..

[70] Smolin G. (1989). Cellular response to inflammation at the limbus. Eye.

[71] Seyed-Safi A.G., Daniels J.T. (2020). The limbus: Structure and function. Exp. Eye Res..

[72] Kempuraj D., Thangavel R., Natteru P.A., Selvakumar G.P., Saeed D., Zahoor H., Zaheer S., Iyer S.S., Zaheer A. (2016). Neuroinflammation Induces Neurodegeneration. J. Neurol. Neurosurg. Spine.

[73] Zhang Z., Kurashima Y. (2021). Two Sides of the Coin: Mast Cells as a Key Regulator of Allergy and Acute/Chronic Inflammation. Cells.

[74] Elieh Ali Komi D., Wohrl S., Bielory L. (2020). Mast Cell Biology at Molecular Level: A Comprehensive Review. Clin. Rev. Allergy Immunol..

[75] Cook E.B., Stahl J.L., Barney N.P., Graziano F.M. (2001). Ocular mast cells. Characterization in normal and disease states. Clin. Rev. Allergy Immunol..

[76] Levin L.A., Albert D.M., Johnson D. (1993). Mast cells in human optic nerve. Investig. Ophthalmol. Vis. Sci..

[77] Cho W., Mittal S.K., Elbasiony E., Chauhan S.K. (2020). Activation of ocular surface mast cells promotes corneal neovascularization. Ocul. Surf..

[78] Irani A.M. (2008). Ocular mast cells and mediators. Immunol. Allergy Clin. N. Am..

[79] Liu J., Li Z. (2021). Resident Innate Immune Cells in the Cornea. Front. Immunol..

[80] Micera A., Jirsova K., Esposito G., Balzamino B.O., Di Zazzo A., Bonini S. (2020). Mast Cells Populate the Corneoscleral Limbus: New Insights for Our Understanding of Limbal Microenvironment. Investig. Ophthalmol. Vis. Sci..

[81] Kempuraj D., Selvakumar G.P., Thangavel R., Ahmed M.E., Zaheer S., Raikwar S.P., Iyer S.S., Bhagavan S.M., Beladakere-Ramaswamy S., Zaheer A. (2017). Mast Cell Activation in Brain Injury, Stress, and Post-traumatic Stress Disorder and Alzheimer’s Disease Pathogenesis. Front. Neurosci..

[82] Dong H., Zhang X., Qian Y. (2014). Mast cells and neuroinflammation. Med. Sci. Monit. Basic Res..

[83] Tsai M., Grimbaldeston M., Galli S.J. (2011). Mast cells and immunoregulation/immunomodulation. Adv. Exp. Med. Biol..

[84] Church M.K., McGill J.I. (2002). Human ocular mast cells. Curr. Opin. Allergy Clin. Immunol..

[85] Patel D., Sarala N., Datti N.P. (2018). Topical Olopatadine Hydrochloride versus Ketotifen Fumarate for Allergic Conjunctivitis. J. Ophthalmic. Vis. Res..

[86] Mello-Bosnic C., Gimenes A.D., Oliani S.M., Gil C.D. (2018). Treatment with galectin-1 eye drops regulates mast cell degranulation and attenuates the severity of conjunctivitis. Eur. J. Pharmacol..

[87] Elieh Ali Komi D., Rambasek T., Bielory L. (2018). Clinical implications of mast cell involvement in allergic conjunctivitis. Allergy.

[88] Bielory L., Kempuraj D., Theoharides T. (2002). Topical immunopharmacology of ocular allergies. Curr. Opin. Allergy Clin. Immunol..

[89] Kempuraj D., Huang M., Kandere K., Boucher W., Letourneau R., Jeudy S., Fitzgerald K., Spear K., Athanasiou A., Theoharides T.C. (2002). Azelastine is more potent than olopatadine in inhibiting interleukin-6 and tryptase release from human umbilical cord blood-derived cultured mast cells. Ann. Allergy Asthma Immunol..

[90] Mounsey A.L., Gray R.E. (2016). Topical Antihistamines and Mast Cell Stabilizers for Treating Allergic Conjunctivitis. Am. Fam. Physician.

[91] Cho W., Mittal S.K., Elbasiony E., Chauhan S.K. (2021). Spatial Distribution of Mast Cells Regulates Asymmetrical Angiogenesis at the Ocular Surface. Am. J. Pathol..

[92] Sahu S.K., Mittal S.K., Foulsham W., Li M., Sangwan V.S., Chauhan S.K. (2018). Mast Cells Initiate the Recruitment of Neutrophils Following Ocular Surface Injury. Investig. Ophthalmol. Vis. Sci..

[93] Wulff B.C., Wilgus T.A. (2013). Mast cell activity in the healing wound: More than meets the eye?. Exp. Dermatol..

[94] Mukai K., Tsai M., Saito H., Galli S.J. (2018). Mast cells as sources of cytokines, chemokines, and growth factors. Immunol. Rev..

[95] Komi D.E.A., Khomtchouk K., Santa Maria P.L. (2020). A Review of the Contribution of Mast Cells in Wound Healing: Involved Molecular and Cellular Mechanisms. Clin. Rev. Allergy Immunol..

[96] Conti P., Caraffa A., Ronconi G., Kritas S.K., Mastrangelo F., Tettamanti L., Frydas I., Theoharides T.C. (2018). Mast cells participate in allograft rejection: Can IL-37 play an inhibitory role?. Inflamm. Res..

[97] Strattan E., Hildebrandt G.C. (2021). Mast Cell Involvement in Fibrosis in Chronic Graft-Versus-Host Disease. Int. J. Mol. Sci..

[98] Crivellato E., Nico B., Ribatti D. (2008). Mast cells and tumour angiogenesis: New insight from experimental carcinogenesis. Cancer Lett..

[99] Ng M.F. (2010). The role of mast cells in wound healing. Int. Wound J..

[100] Metcalfe D.D., Baram D., Mekori Y.A. (1997). Mast cells. Physiol. Rev..

[101] Barrientez B., Nicholas S.E., Whelchel A., Sharif R., Hjortdal J., Karamichos D. (2019). Corneal injury: Clinical and molecular aspects. Exp. Eye Res..

[102] Wilson S.E. (2020). Corneal wound healing. Exp. Eye Res..

[103] Wilson S.E. (2020). Corneal myofibroblasts and fibrosis. Exp. Eye Res..

[104] Zhu X.R., Du J.H. (2018). Autophagy: A potential target for the treatment of intraocular neovascularization. Int. J. Ophthalmol..

[105] Mohan R.R., Tovey J.C., Sharma A., Schultz G.S., Cowden J.W., Tandon A. (2011). Targeted decorin gene therapy delivered with adeno-associated virus effectively retards corneal neovascularization in vivo. PLoS ONE.

[106] Vlasov A., Ryan D.S., Ludlow S., Coggin A., Weichel E.D., Stutzman R.D., Bower K.S., Colyer M.H. (2017). Corneal and Corneoscleral Injury in Combat Ocular Trauma from Operations Iraqi Freedom and Enduring Freedom. Mil. Med..

[107] Kempuraj D., Ahmed M.E., Selvakumar G.P., Thangavel R., Dhaliwal A.S., Dubova I., Mentor S., Premkumar K., Saeed D., Zahoor H. (2019). Brain Injury-Mediated Neuroinflammatory Response and Alzheimer’s Disease. Neuroscientist.

[108] DePalma R.G., Hoffman S.W. (2018). Combat blast related traumatic brain injury (TBI): Decade of recognition; promise of progress. Behav. Brain Res..

[109] Edwards G., Zhao J., Dash P.K., Soto C., Moreno-Gonzalez I. (2020). Traumatic Brain Injury Induces Tau Aggregation and Spreading. J. Neurotrauma.

[110] Kokiko-Cochran O.N., Godbout J.P. (2018). The Inflammatory Continuum of Traumatic Brain Injury and Alzheimer’s Disease. Front. Immunol..

[111] Hay J.R., Johnson V.E., Young A.M., Smith D.H., Stewart W. (2015). Blood-Brain Barrier Disruption Is an Early Event That May Persist for Many Years after Traumatic Brain Injury in Humans. J. Neuropathol. Exp. Neurol..

[112] Cockerham G.C., Lemke S., Rice T.A., Wang G., Glynn-Milley C., Zumhagen L., Cockerham K.P. (2014). Closed-globe injuries of the ocular surface associated with combat blast exposure. Ophthalmology.

[113] Cockerham G.C., Goodrich G.L., Weichel E.D., Orcutt J.C., Rizzo J.F., Bower K.S., Schuchard R.A. (2009). Eye and visual function in traumatic brain injury. J. Rehabil. Res. Dev..

[114] Lee C.J., Felix E.R., Levitt R.C., Eddy C., Vanner E.A., Feuer W.J., Sarantopoulos C.D., Galor A. (2018). Traumatic brain injury, dry eye and comorbid pain diagnoses in US veterans. Br. J. Ophthalmol..

[115] Armstrong R.A. (2018). Visual problems associated with traumatic brain injury. Clin. Exp. Optom..

[116] Gupta S., Kamil S., Sinha P.R., Rodier J.T., Chaurasia S.S., Mohan R.R. (2021). Glutathione is a potential therapeutic target for acrolein toxicity in the cornea. Toxicol. Lett..

[117] Rai D.K., Sharma B. (2007). Carbofuran-induced oxidative stress in mammalian brain. Mol. Biotechnol..

[118] Charkoftaki G., Jester J.V., Thompson D.C., Vasiliou V. (2018). Nitrogen mustard-induced corneal injury involves the sphingomyelin-ceramide pathway. Ocul. Surf..

[119] Gupta R.C. (1994). Carbofuran toxicity. J. Toxicol. Environ. Health.

[120] Goswami D.G., Tewari-Singh N., Agarwal R. (2016). Corneal toxicity induced by vesicating agents and effective treatment options. Ann. N. Y. Acad. Sci..

[121] Achanta S., Jordt S.E. (2021). Toxic effects of chlorine gas and potential treatments: A literature review. Toxicol. Mech. Methods.

[122] Govier P., Coulson J.M. (2018). Civilian exposure to chlorine gas: A systematic review. Toxicol. Lett..

[123] Bhattacharya S.K., Hom G.G., Fernandez C., Hom L.G. (2007). Ocular effects of exposure to industrial chemicals: Clinical management and proteomic approaches to damage assessment. Cutan. Ocul. Toxicol..

[124] Schwenk M. (2018). Chemical warfare agents. Classes and targets. Toxicol. Lett..

[125] Choi S.I., Kim E.K. (2016). Autophagy in granular corneal dystrophy type 2. Exp. Eye Res..

[126] Shivakumar S., Panigrahi T., Shetty R., Subramani M., Ghosh A., Jeyabalan N. (2018). Chloroquine Protects Human Corneal Epithelial Cells from Desiccation Stress Induced Inflammation without Altering the Autophagy Flux. Biomed. Res. Int..

[127] Martinez-Chacon G., Vela F.J., Campos J.L., Abellan E., Yakhine-Diop S.M.S., Ballestin A. (2020). Autophagy modulation in animal models of corneal diseases: A systematic review. Mol. Cell Biochem..

[128] Shetty R., Sharma A., Pahuja N., Chevour P., Padmajan N., Dhamodaran K., Jayadev C., Nuijts R.M.M.A., Ghosh A., Nallathambi J. (2017). Oxidative stress induces dysregulated autophagy in corneal epithelium of keratoconus patients. PLoS ONE.

[129] Cui W., Wu X., Feng D., Luo J., Shi Y., Guo W., Liu H., Wang Q., Wang L., Ge S. (2021). Acrolein Induces Systemic Coagulopathy via Autophagy-dependent Secretion of von Willebrand Factor in Mice after Traumatic Brain Injury. Neurosci. Bull..

[130] Zhang Y.B., Li S.X., Chen X.P., Yang L., Zhang Y.G., Liu R., Tao L.Y. (2008). Autophagy is activated and might protect neurons from degeneration after traumatic brain injury. Neurosci. Bull..

[131] Au A.K., Aneja R.K., Bayir H., Bell M.J., Janesko-Feldman K., Kochanek P.M., Clark R.S.B. (2017). Autophagy Biomarkers Beclin 1 and p62 are Increased in Cerebrospinal Fluid after Traumatic Brain Injury. Neurocrit. Care.

[132] Sarkar C., Zhao Z., Aungst S., Sabirzhanov B., Faden A.I., Lipinski M.M. (2014). Impaired autophagy flux is associated with neuronal cell death after traumatic brain injury. Autophagy.

[133] Sarkar C., Jones J.W., Hegdekar N., Thayer J.A., Kumar A., Faden A.I., Kane M.A., Lipinski M.M. (2020). PLA2G4A/cPLA2-mediated lysosomal membrane damage leads to inhibition of autophagy and neurodegeneration after brain trauma. Autophagy.

[134] Yin Y., Sun G., Li E., Kiselyov K., Sun D. (2017). ER stress and impaired autophagy flux in neuronal degeneration and brain injury. Ageing Res. Rev..

